# Mapping the undergraduate medical curriculum of the Charité Berlin against the National Competence-Based Catalogue of Learning Objectives (NKLM 2.0)

**DOI:** 10.3205/zma001770

**Published:** 2025-09-15

**Authors:** Tabea Theurich, Ylva Holzhausen, Olaf Ahlers, Harm Peters

**Affiliations:** 1 Charité – Universitätsmedizin Berlin, Dieter Scheffner Center for Medical Education and Educational Research, Dean’s Office of Study Affairs, Berlin, Germany; 2 Brandenburg Medical School Theodor Fontane, Faculty of Health Sciences Brandenburg, Institute of Research in Health Sciences Education, Neuruppin, Germany; 3 Charité – Universitätsmedizin Berlin, Institute of Medical Informatics, Berlin, Germany

**Keywords:** National Competence-Based Catalogue of Learning Objectives for Undergraduate Medical Education (NKLM), curriculum mapping, undergraduate medical program, competency-based education

## Abstract

**Background:**

The National Competence-Based Catalogue of Learning Objectives (NKLM) aims to set standards for undergraduate medical education programs in Germany. The NKLM 2.0 is currently under revision, and medical faculties have been invited to evaluate this version. This study maps the learning objectives of the Modular Curriculum of Medicine (MCM) of the Charité Berlin to the NKLM 2.0 items using approaches based to those of a previous mapping of NKLM 1.0 to allow comparison.

**Methods:**

A two-step process was used to determine coverage. First, the MCM learning objectives were mapped to the NKLM 2.0 items using the LOOOP curriculum management platform. Next, the degree of coverage of the NKLM 2.0 by the MCM was calculated via three quantitative approaches and one qualitative approach (content comparison). Finally, the results of NKLM 2.0 coverage were compared with the results of the previous NKLM 1.0 mapping.

**Results:**

The mapping process identified 11,879 matches between 4,396 MCM learning objectives and 2,813 NKLM 2.0 items. The degree of NKLM 2.0 coverage, based on a content comparison approach increased to 52% compared with 41% for the NKLM 1.0. While some NKLM 2.0 chapters showed considerable increases (VII +25%, VIII +20%), others showed relevant decreases (V -8%, VI -8%).

**Discussion:**

The coverage of the NKLM 2.0 by MCM learning objectives has improved compared with the NKLM 1.0. However, the low level of coverage by the MCM of only 52% indicates that the content of future revisions of the NKLM needs to be reduced considerably; otherwise, the content cannot feasibly be taught and learned within the regulatory framework for undergraduate medical education in Germany.

## Zusammenfassung

**Hintergrund:**

Der Nationale Kompetenzbasierte Lernzielkatalog Medizin (NKLM) soll Standards für die medizinische Ausbildung in Deutschland setzen. Derzeit wird der NKLM 2.0 überarbeitet und die medizinischen Fakultäten wurden aufgerufen, diese Version zu evaluieren. In dieser Studie werden die Lernziele des Modellstudiengangs Medizin (MSM) der Charité Berlin gegen die Items des NKLM 2.0 kartiert. Dabei kommen Verfahren, wie bei der vorherigen Kartierung des NKLM 1.0 zum Einsatz, um einen Vergleich zu ermöglichen.

**Methoden:**

Ein zweistufiger Prozess wurde zur Ermittlung des curricularen Abdeckungsgrads verwendet. Zunächst wurden die Lernziele des MSM mithilfe der Curriculum-Management-Plattform LOOOP den Items des NKLM 2.0 zugeordnet. Anschließend wurde der Abdeckungsgrad des NKLM 2.0 durch den MSM mithilfe von drei quantitativen Ansätzen und einem qualitativen Ansatz (Inhaltsvergleich) berechnet. Abschließend wurden die Ergebnisse der NKLM 2.0-Abdeckung mit den Ergebnissen der vorherigen NKLM 1.0-Kartierung verglichen.

**Ergebnisse:**

Der Kartierungs-Prozess ergab 11.879 Übereinstimmungen zwischen 4.396 MSM-Lernzielen und 2.813 NKLM 2.0-Items. Der Abdeckungsgrad des NKLM 2.0, basierend auf dem qualitativen Inhaltsvergleich, erhöhte sich auf 52%, verglichen mit 41% für den NKLM 1.0. Während einige NKLM 2.0-Kapitel deutliche Steigerungen des Abdeckungsgrades aufwiesen (VII +25%, VIII +20%), waren in anderen Kapiteln relevante Rückgänge zu verzeichnen (V -8%, VI -8%).

**Diskussion:**

Im Vergleich zum NKLM 1.0 hat sich die Abdeckung des NKLM 2.0 durch MSM-Lernziele verbessert. Der nach wie vor niedrige Abdeckungsgrad von nur 52% legt jedoch nahe, dass der Inhalt des NKLM im Zuge zukünftiger Revisionen deutlich reduziert werden sollte. Andernfalls wird er nicht realistisch im gegebenen Rahmen für die medizinischen Ausbildung in Deutschland umgesetzt werden können.

## 1. Introduction

National outcome frameworks represent a cornerstone of competency-based medical education (CBME) in countries such as Canada, the United Kingdom, Switzerland and, more recently, Germany [1], [2], [3]. These frameworks operationalize and harmonize standards for core knowledge, skills and attitudes that graduates at the national level should acquire over the course of a medical training program [4], [5], [6]. The National Competence-Based Catalogue of Learning Objectives (NKLM) of the Association of Medical Faculties in Germany (MFT) aims to set standards for undergraduate medical education programs in Germany. The first version (NKLM 1.0) was published in 2015, and a substantially revised version (NKLM 2.0) was released in 2021 [https://nklm.de/zend/menu]. Medical faculties in Germany have been invited by the MFT to evaluate the usability and the feasibility of the NKLM 2.0 in their context with the goal of providing information that can be used to support the further development of the NKLM. The purpose of this study is to follow this call by mapping the Modular Curriculum of Medicine (MCM) of the Charité Berlin against the NKLM 2.0 to estimate coverage by the revised national standard by employing approaches analogous to those used previously for mapping the NKLM 1.0, thereby enabling comparisons.

In previous work, our group mapped the learning objectives of the MCM, the undergraduate medical program of our medical faculty, onto the NKLM 1.0 via the in-house LOOOP curriculum management platform version, called LLP [https://lernziele.charite.de/zend/]. The MCM is an integrated, competency-based curriculum that is in line with the competency-based framework of the NKLM and the intended reform of the German licensing regulations for physicians [https://www.gesetze-im-internet.de/_appro_2002/BJNR240500002.html]. The design and implementation of the MCM followed an outcome-based approach by building on a competency framework developed at the Charité [7]. This framework, like the NKLM, contains specified knowledge, skills and competence domains. The MCM learning objectives were defined through a faculty-wide process that resulted in the identification and setting of specific learning objectives for all teaching sessions. In particular, this process was conducted in a continuous co-design process that involved faculty from different disciplines as well as educators and students [7], [8]. This made it possible to limit the breadth and depth of these learning objectives to the time available for different teaching formats, including time for preparation and follow-up tasks for students.

In our previous work, we assessed the relative coverage of the NKLM 1.0 by the MCM using four approaches. These included three primarily quantitative methods (single match, multiple match, and subordinate match) and one qualitative approach (content comparison) to provide complementary perspectives on curriculum coverage [9]. While the quantitative methods indicated the breadth of NKLM coverage, simple numerical matching failed to capture the depth and quality of content, which led to a systematic overestimation of actual coverage (e.g., 73% with the single-match approach). In contrast, the qualitative content comparison provided a more accurate estimation of both the breadth and depth of coverage and revealed that the MCM learning objectives covered only 41% of the NKLM 1.0 [9]. To date, this remains the only published study that has mapped an entire curriculum against the NKLM 1.0. Other reports have focused on specific methodological aspects, such as the subordinate match approach, or particular content areas, such as research competencies [4], [10], [11].

The NKLM 2.0 represents a substantially revised version of the NKLM 1.0. To date, no study has reported the mapping of an entire undergraduate medical curriculum of a German medical faculty against the NKLM 2.0. Instead, some results of partial mappings against the new NKLM 2.0 have been published. For example, Plange et al. reported the extent to which ophthalmology is represented in the NKLM 2.0 [12]. The German Radiological Society (DRG) developed a radiology curriculum that contributed to the NKLM 2.0 [13]. The Hannover Medical School evaluated part of the NKLM 2.0 learning objectives by matching them on the basis of preexisting mappings of the NKLM 1.0 and evaluating them [11].

The primary aim of this study was to map the learning objectives of the MCM against the items in NKLM 2.0 using a two-step process with four approaches to derive the degree of coverage following a methodology similar to our previous NKLM 1.0 mapping [9]. The second aim was to compare MCM coverage between the NKLM 1.0 and the NKLM 2.0. The findings of this study are intended to inform the development of future versions of the NKLM and complement the ongoing NKLM 2.0 revision process led by the MFT and its working groups.

## 2. Methods

### 2.1. Setting

The study was conducted at the Dieter Scheffner Center for Medical Education and Educational Research at the Charité – Universitätsmedizin Berlin (Charité) from December 2022 to April 2024 using the LOOOP curriculum management platform [14]. The NKLM 2.0 mapping followed the previously established NKLM 1.0 methodology to enable direct comparison between both studies.

### 2.2. Structure of the MCM

The MCM spans six years including a final clerkship year. The first five years are divided into 40 modules. Four modules have no learning objectives as they are compulsory or repetition modules. These years include a total of 3,580 teaching hours consisting of 45 minutes each with a total of 4,396 learning objectives. No specific learning objectives are implemented for the final clerkship year. The MCM objectives are organized into three hierarchical levels and include competency-based program outcomes (level 1), overarching module-level objectives (level 2), and teaching session-specific objectives (level 3). The objectives are categorized by knowledge, skills, and attitudes, and they follow a LOOOP-specific taxonomy based on Miller’s pyramid [15] and Bloom’s taxonomy [16]. The MCM learning objectives, which are mandatory for all students, cover approximately 80% of the program's teaching time.

### 2.3. Structure of the NKLM

The NKLM 2.0 consists of multiple chapters. Chapters I–IV provide introductory and explanatory content; chapter V outlines 167 “consultation occasions”; and chapter VI lists 598 “diseases” grouped into 11 categories. Chapters VII and VIII follow a three-tier structure that comprises 97 “competencies”, 340 “subcompetencies”, and 2,048 “learning objectives” with increasing granularity. Because the NKLM 2.0 has different sections, we use the term "NKLM items" in this article to collectively refer to consultation occasions, diseases, and learning objectives. The NKLM aims to cover approximately 70-80% of undergraduate medical education programs in Germany.

The NKLM 2.0 employs two systems to define the depth of learning objectives on the basis of LOOOP’s taxonomy (see table 1 (Tab. 1)). Chapters V and VI use letter-coded descriptors for different aspects of consultation occasions or diseases, whereas chapters VII and VIII assign numerical codes to competency depths. The NKLM 2.0 chapters interact with one another via approximately 15,000 cross-references. These were not considered in this mapping process as the additional workload would have been disproportionately high.

The structure of the NKLM 1.0 was revised during the development of the NKLM 2.0. Table 2 (Tab. 2) compares the corresponding chapters in both versions [https://nklm.de/zend/menu].

### 2.4. Description of the mapping process

The mapping was conducted using the LOOOP platform that allows to align the hierarchical structures of the MCM learning objectives and the NKLM 2.0 items to ensure a systematic and transparent approach.

The MCM learning objectives were mapped to the NKLM 2.0 items via a two-step process. First, level 3 MCM objectives were matched to NKLM 2.0 items. Coverage was then assessed using four methods similar to those established by Gulbis et al. [9] for the NKLM 1.0. Mapping followed a dual-control principle (four-eyes principle) to enhance reliability and minimize bias. Discrepancies were resolved through discussion until consensus was reached. The mapping team comprised a senior MCM student (author TT) and the head of the Dieter Scheffner Center for Medical Education (author HP), who played a key role in the implementation of the MCM and prior NKLM 1.0 mapping.

The NKLM 2.0 was mapped against the MCM learning objectives from the summer semester of 2022 with a focus on the most detailed level of both catalogues to allow a detailed analysis of their alignment. The mapping included 2,813 NKLM items and 4,396 MCM objectives. Only minor changes were observed in the MCM objectives compared with those from the summer semester of 2018, which were used for the NKLM 1.0 mapping.

#### 2.4.1. Step 1 – mapping of MCM to NKLM 2.0 items

The mapping followed a three-stage process. First, TT conducted a forward-mapping approach by matching the MCM learning objectives to the NKLM 2.0 items using keyword searches and prior NKLM 1.0 mappings in the LOOOP platform. The guiding principles were iteratively refined throughout the process. Second, HP reviewed each mapping for accuracy. Finally, a backward-mapping approach with a focus on gaps in NKLM 2.0 coverage identified additional NKLM items that aligned with the MCM objectives. The LLP platform was used to search for potential matches.

To ensure consistency, transparency, and accuracy, the mapping adhered to the following principles:

1. A match between an MCM learning objective and an NKLM 2.0 item was based on content overlap regardless of the degree of conformity.
2. Consultation occasions were not mapped to possible underlying diseases unless explicitly stated.
3. Only explicit statements in the learning objectives were considered part of the mapping process. In the case of bedside teaching courses in the MCM, information on the covered diseases was drawn from the LLP [https://lernziele.charite.de/zend/].
4. The descriptor “G” for basic knowledge was mapped only from the fifth semester onward to reinforce basic sciences in clinical contexts.
5. Consultation occasions or diseases were mapped without a descriptor if none applied.
6. The mapped learning objectives were assigned to the same category (“knowledge” or “skills”) based on LOOOP’s taxonomy.

#### 2.4.2. Step 2 – deriving the relative coverage of the NKLM by the MCM

In step 2, four approaches were chosen that build on established curriculum mapping methodologies [17], [18] and our previous work with the NKLM 1.0 [9]. The combination of primarily quantitative and qualitative approaches was selected to provide complementary perspectives on curriculum coverage by the MCM. Attachment 1 visualizes the three quantitative mapping approaches to allow better comprehension. The qualitative content comparison approach provides deeper insights into the alignment of the content in terms of both the scope and depth of coverage.

The mapping results from step 1 were exported into data files, and NKLM 2.0 coverage by the MCM was calculated using four approaches:

1. *Single match:* An NKLM item was considered covered if at least one MCM objective was matched.
2. *Multiple match: *An NKLM item was considered covered if at least three MCM objectives were matched.
3. *Subordinate match: *The coverage of an NKLM objective at level 2 was derived on the basis of the percentage of its subordinate objectives (level 3) that were covered using the single-match method.
4. *Content comparison:* The qualitative approach was divided into two variants on the basis of the structure of the NKLM 2.0. Both variants relied on the same scale to indicate the degree of coverage:
  0=0%, 1=1%-20%, 2=21%-40%, 3=41%-60%, 4=61%-80%, 5=81%-100%.
5.
  1. *Variant 1 (chapters V & VI): *The depth of competency was scored with “W” (knowledge) assigned 1 point and “H” (skills) assigned 2 points. Each NKLM 2.0 item had a maximum of 11 points based on descriptors (D, T, N, P, M=2 points each, G=1 point). The total points covered by the MCM objectives were converted into a scale from 0 to 5.
  2. *Variant 2 (chapters VII & VIII):* Each rater assigned coverage scores (0–5) based on content coverage. If the MCM objectives did not match the NKLM competency depth, the score was reduced by at least one point. Full consensus retained the chosen score, good consensus (difference of 1) was rounded up, and no consensus (difference >1) led to discussion and agreement on a final score.

The mapping process spanned 16 months and required approximately 1,500 hours of work by TT and HP.

### 2.5. Statistical analysis

Statistical analysis was performed using IBM SPSS Statistics 29.0.1.1 and Microsoft Excel 16.83. The descriptive statistics included absolute and relative numbers and means. Weighted means were calculated by multiplying each mean by its weight, which was derived from the proportion of learning objectives in a subchapter relative to the entire chapter. The sum of these weighted values represented the final weighted mean. Interrater reliability for the qualitative approach in chapters VII and VIII was assessed using Cohen’s kappa. It was not calculated for chapters V and VI due to their numerical-based analysis. Coverage comparisons between NKLM 1.0 and 2.0 were based on the corresponding chapters, as detailed in table 2 (Tab. 2).

## 3. Results

### 3.1. Step 1– mapping the MCM against the NKLM items

**Figure 1:**
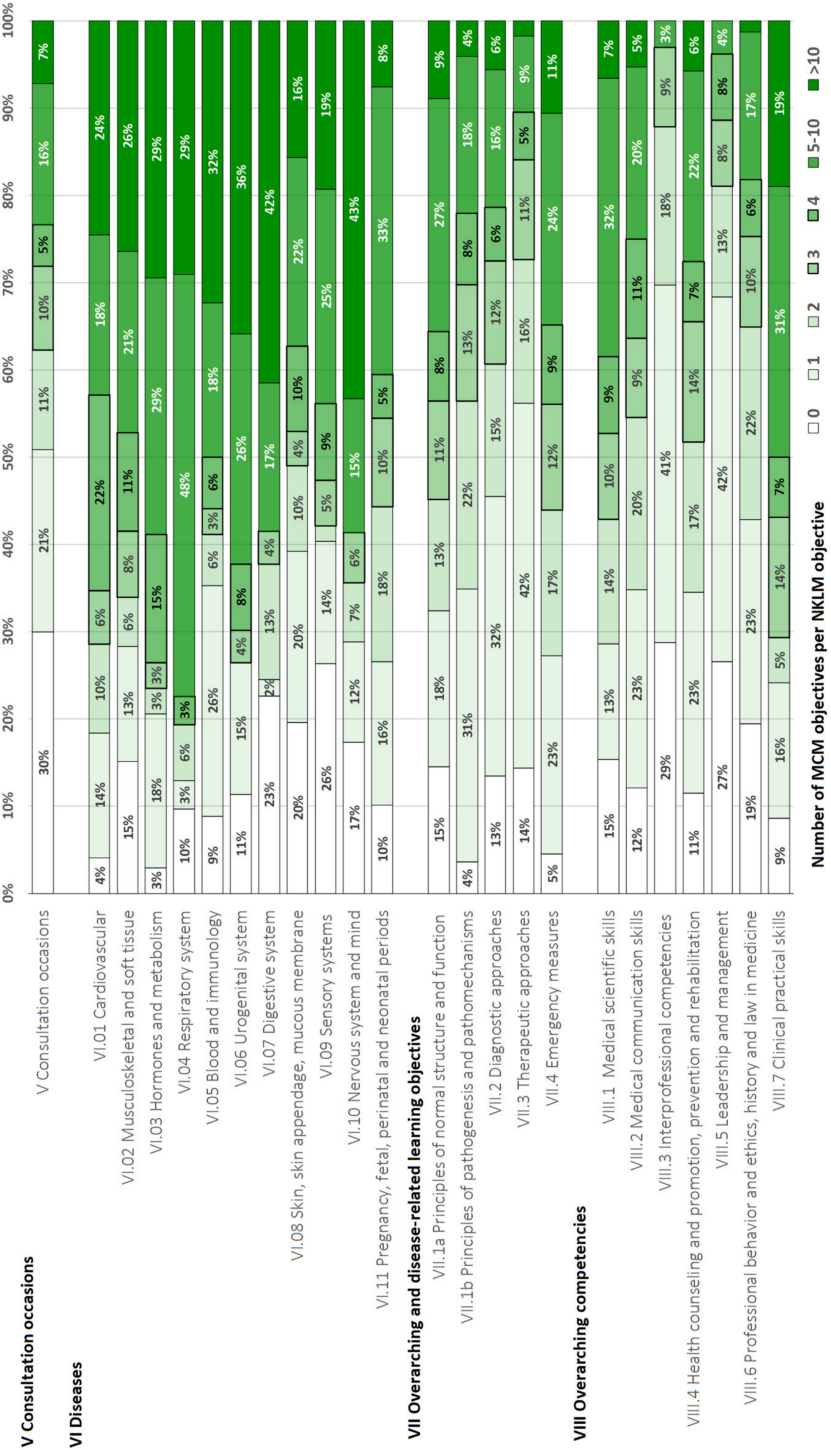
Overview of the relative contribution of the Modular Curriculum of Medicine (MCM) learning objectives mapped to the National Competence-Based Catalogue of Learning Objectives (NKLM) 2.0 items, categorized from 0 to more than 10 mappings per item (see colour legend in the lower part of the figure).

The MCM learning objectives were mapped 11,879 times to 2,813 NKLM 2.0 items using 98% of the 4,396 MCM objectives. Table 3 (Tab. 3) presents the numerical results, and figure 1 (Fig. 1) presents the mappings per NKLM item across chapters. On average, 4.2 MCM objectives were mapped per NKLM item; this number increased to 5.0 when unmapped items were excluded. A total of 417 NKLM items (15%) had no match. The highest rate of unmatched items was in chapter V (consultation occasions) (30%), and the lowest was in chapter VII (overarching and disease-related learning objectives) (12%).

### 3.2. Step 2 – deriving the relative coverage of the NKLM by the MCM

**Figure 2:**
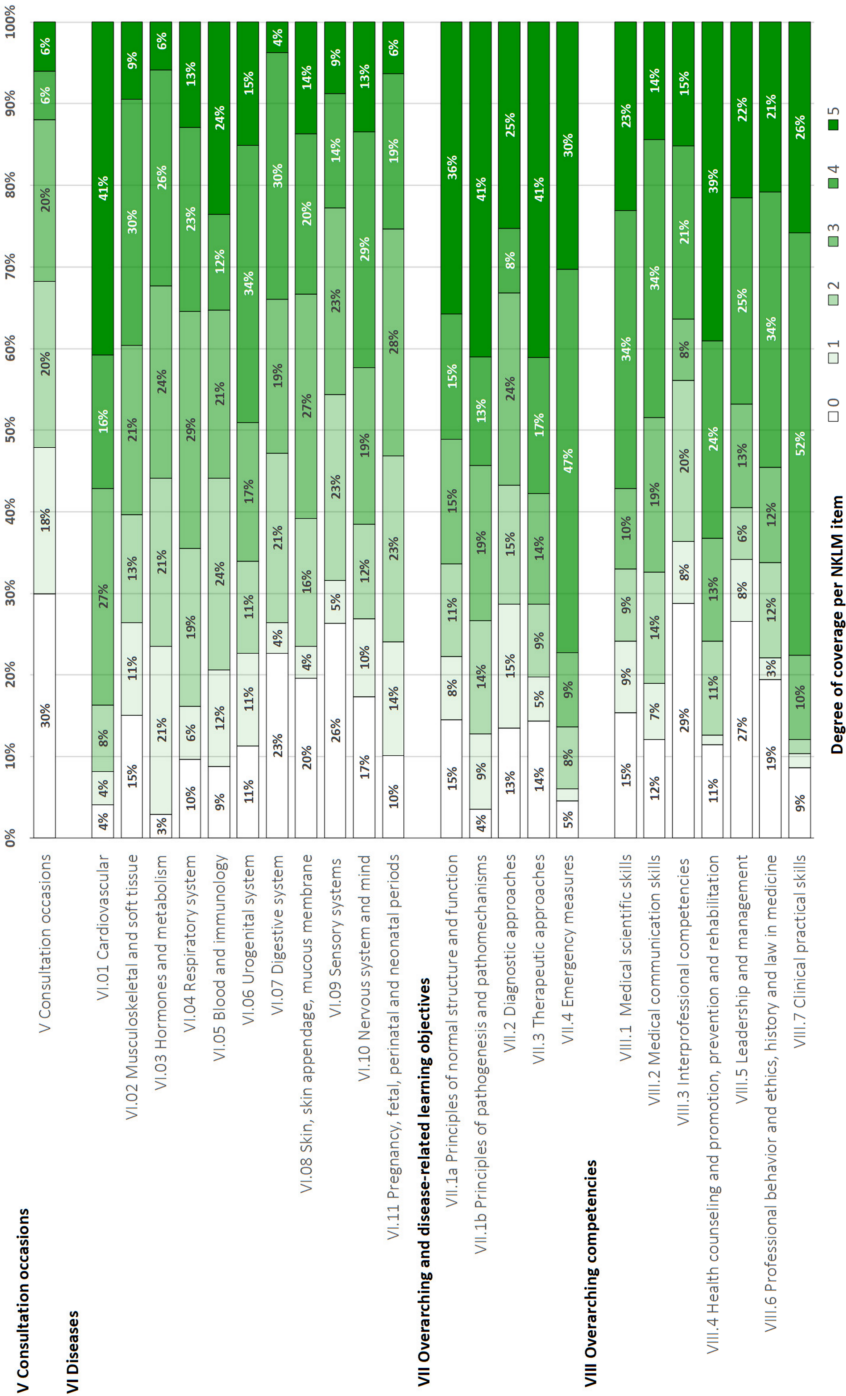
Relative degree of coverage in the National Competence-Based Catalogue of Learning Objectives 2.0 (NKLM) chapters by Modular Curriculum of Medicine (MCM) learning objectives based on the qualitative content comparison mapping approach. Ratings range from 0 (no coverage) to 5 (>80-100% coverage) and represent the relative coverage in percentage form (see colour legend in the lower part of the figure).

Table 4 (Tab. 4) and figure 2 (Fig. 2) present the results of the qualitative content comparison for the NKLM 2.0 and its chapters. Interrater reliability was strong for chapters VII and VIII (Cohen's kappa=0.68). The raters initially reached full consensus in 75% of cases (n=1,320), good consensus in 17% (n=298), and no consensus in 8% (n=149).

The degree of qualitative coverage of the NKLM 2.0 items by the MCM learning objectives varied by chapter, with the lowest in chapter V (consultation occasions) and the highest in subchapter VII.4 (emergency measures).

**Figure 3:**
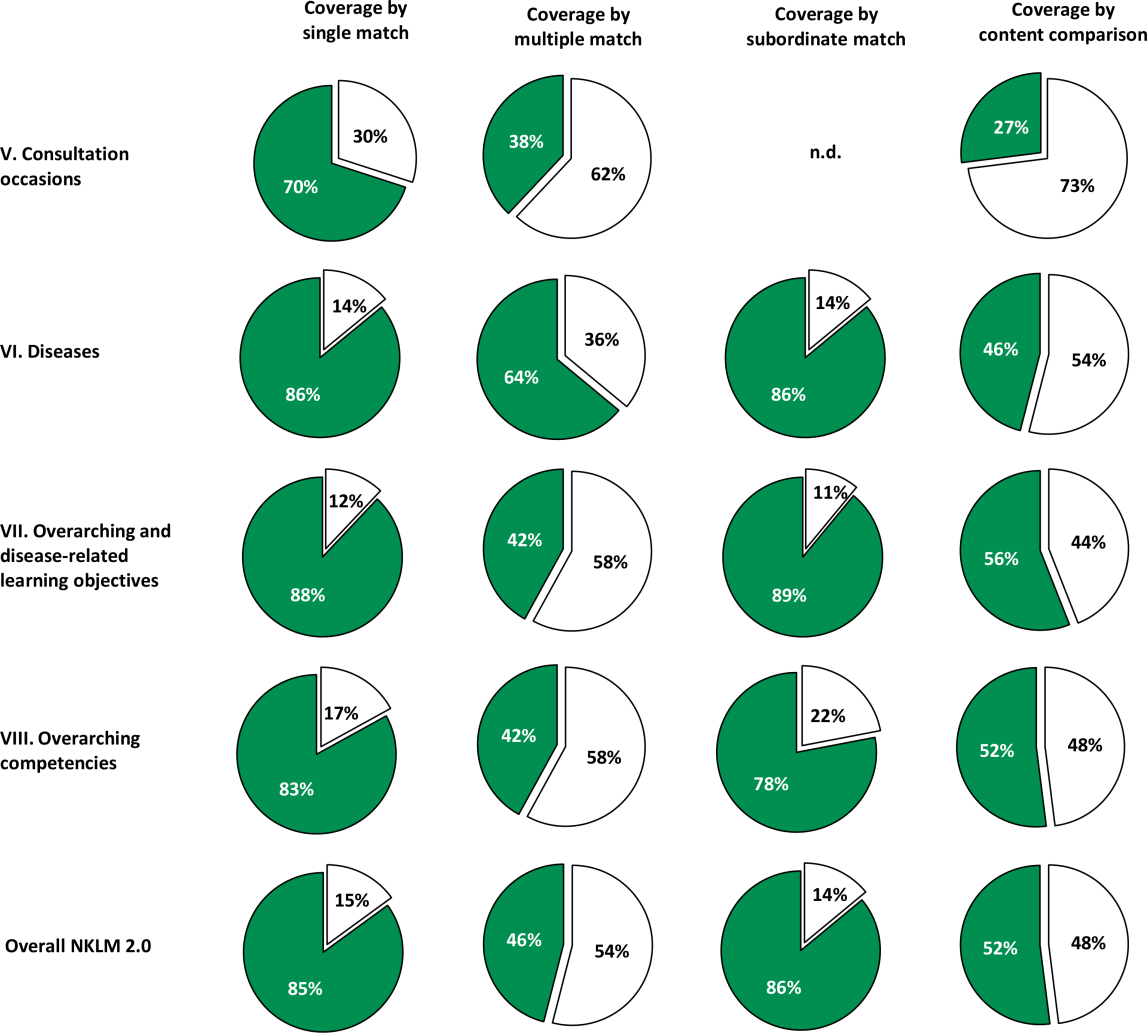
Overview of the degree of coverage exhibited by the National Competence-Based Catalogue of Learning Objectives 2.0 (NKLM) overall and by chapters based on mappings of Modular Curriculum of Medicine (MCM) learning objectives across the four mapping approaches: for each single match, multiple match, subordinate match and the qualitative content comparison approach (n.d.=not determined). The green areas indicate the estimated degree of coverage.

Figure 3 (Fig. 3) visually presents the degree of NKLM 2.0 coverage across the four approaches. Table 5 (Tab. 5) details the comparisons by the methods used. The single-match and subordinate-match approaches showed the highest degree of coverage (85% and 86%), whereas the multiple-match approach showed the lowest degree of coverage (46%). The qualitative content comparison approach resulted in 52% coverage of the NKLM by the MCM.

### 3.3. Comparison of the coverage of the NKLM 1.0 and the NKLM 2.0 by the MCM

Compared with our previous mapping of the NKLM 1.0, there were some changes in the number of MCM learning objectives and NKLM items for this study. The MCM objectives decreased slightly from 4,490 (2018) to 4,396 (2022) (-3%), whereas the number of NKLM items increased from 2,105 to 2,813 (+34%). The largest growth occurred in chapter VII (overarching and disease-related learning objectives) (+507 items).

Table 6 (Tab. 6) compares the degree of coverage of the NKLM 1.0 and 2.0 across chapters. The NKLM 2.0 had higher MCM coverage across all four approaches. The largest increase was in the subordinate-match approach (+19%), whereas the smallest increase was in the multiple-match approach (+4%). The qualitative content comparison approach showed an 11% overall increase, with notable gains in chapters VII (+25%) and VIII (+20%) and declines in chapters V and VI (-8% each).

## 4. Discussion

CBME frameworks, such as the NKLM for undergraduate medical education in Germany, aim to ensure that medical graduates are equipped with the necessary competencies – knowledge, skills, and attitudes – that they need to contribute effectively to the care of patients after beginning postgraduate medical training. The present study showed that the substantially revised NKLM 2.0 was only 52% covered when it was mapped to the learning objectives of the competency-based undergraduate medical MCM program at our institution. Below, we discuss the methodology and findings of this mapping study in the context of the literature, particularly our previous work on mapping the MCM against the NKLM 1.0 [9].

The mapping process was greatly facilitated by the specific mapping functionality of the LOOOP platform, which allowed us to build on previous work that mapped the MCM objectives to the NKLM 1.0. Nevertheless, the mapping process remained labour-intensive overall. LOOOP enabled us to map the written NKLM items and MCM learning objectives as the explicit declared curriculum content. Explicitily articulated learning objectives are fundamental to CBME and provide a transparent foundation for constructive alignment between the curriculum that is taught, learned, and assessed to inform all stakeholders, such as medical faculties, teachers, students and curriculum developers [19]. Overall, the three quantitative methods provided a general overview of the NKLM 2.0 items covered, their distribution across chapters, and the number of corresponding MCM objectives without reflecting the extent of content coverage. They generally failed to capture the breadth and depth of the NKLM 2.0 coverage by the learning objectives of the MCM, which can range from very low to complete coverage. Their limitation is akin to estimating the amount of fish in a lake by counting the number of fish without considering variations in size or species. This limitation is important to note as most published estimates of coverage have been based solely on quantitative approaches [4], [10], [20]. As the learning objectives provide a qualitative description of the breadth and depth of the content to be taught, we argue that the calculation of the coverage of a curriculum such as the MCM by the NKLM 2.0 requires a qualitative approach with content comparisons among the objectives to best estimate and describe the degree of content coverage.

Our content-comparison approach showed that, overall, 52% of the NKLM 2.0 items were covered by the MCM learning objectives, indicating a notable improvement over the coverage of the NKLM 1.0. However, coverage varied significantly across chapters, with substantial increases in chapters VII and VIII but decreases in chapters V and VI. These findings regarding the overall coverage of NKLM 2.0 carry implications for future undergraduate medical education programs within the existing regulatory framework in Germany. If the depth and breadth of the NKLM remain at the current level, the duration of the undergraduate program would have to be doubled to 12 years to allow its content to be taught in a feasible manner. Because doubling the duration does not represent a genuine option, ongoing efforts to revise the NKLM should seek to reduce the overall content by approximately 50% on the basis of the qualitative estimation performed in this study.

One reason for the disparity between the scope of the NKLM 2.0 items and the MCM objectives may stem from the manner in which they were developed. The NKLM 2.0 items were largely defined based on the input of various expert groups with discussion that included students or primary care physicians. However, there was no formal limitation on the content to be included. In contrast, the MCM objectives were actually tailored to the reality of the available teaching hours and formats (i.e. content feasibility) in the context of a long, faculty-wide curriculum development process that featured active co-design by medical students [7]. As a result, a part of the proposed content could not be accommodated in the MCM curriculum due to feasibility constraints.

Defining a competency-based framework such as the NKLM is a challenging task for several reasons. The first issue is content validity, or deciding what content to include and, even more challenging, what not to include. Second, if a topic is included, it must involve an appropriate depth of coverage. Challenges arise as the lines between undergraduate and postgraduate medical education are blurry. Third, the breadth of undergraduate medical education stands in sharp contrast to the relative narrowness and depth that characterize practice in the specific discipline in which graduates will ultimately work. Beyond content validity, feasibility must also be taken into account to ensure that the NKLM aligns with the available teaching hours, formats, and student workloads in undergraduate medical education in Germany. Our previous study on the NKLM 1.0 highlighted feasibility concerns as it showed only 41% coverage by the MCM program. The revised NKLM 2.0 shows improved feasibility and reached 52% coverage. However, our findings suggest that a further reduction of content of approximately 50% would be necessary for the NKLM to function as a realistic and feasible CBME framework for undergraduate medical education programs within the German regulatory context.

This study has several limitations. First, a single-center study design was employed. Other German medical universities are invited to report their mapping using a similar qualitative content comparison approach to determine the generalisability of the results of this study to other contexts. The mapping of learning objectives and the calculation of content coverage in this study reflect the shared interpretation of two raters, and larger or different groups may yield varying results. Furthermore, the curriculum mapping process was based on the explicitly declared MCM curriculum. The curriculum that is actually taught may diverge from this situation. Furthermore, cross-references within the NKLM 2.0 were not considered, which may have led to an overestimation of coverage in chapters V and VI as certain descriptors may have been considered to be covered.

In conclusion, this study demonstrates an improvement in NKLM 2.0 coverage by the MCM learning objectives compared with the NKLM 1.0. However, coverage varied across chapters in the NKLM 2.0, with increases in some chapters and decreases in others. The overall level of coverage of the NKLM 2.0 items by the MCM program, which remains low, raises considerable concerns about the feasibility of teaching and learning of the current content of the NKLM 2.0 within the regulatory framework for undergraduate medical education in Germany. The results of this study suggest that the content of the revised future NKLM should be notably reduced to allow for feasible implementation of the curriculum.

## Authors’ ORCIDs

- Tabea Theurich: [0009-0002-9164-4292]
- Ylva Holzhausen: [0000-0001-8710-8257]
- Olaf Ahlers: [0000-0003-1528-7182]
- Harm Peters: [0000-0003-1441-7512]

## Competing interests

The authors declare that they have no competing interests. 

## Supplementary Material

Illustration of the quantitative mapping approaches

## Figures and Tables

**Table 1:**
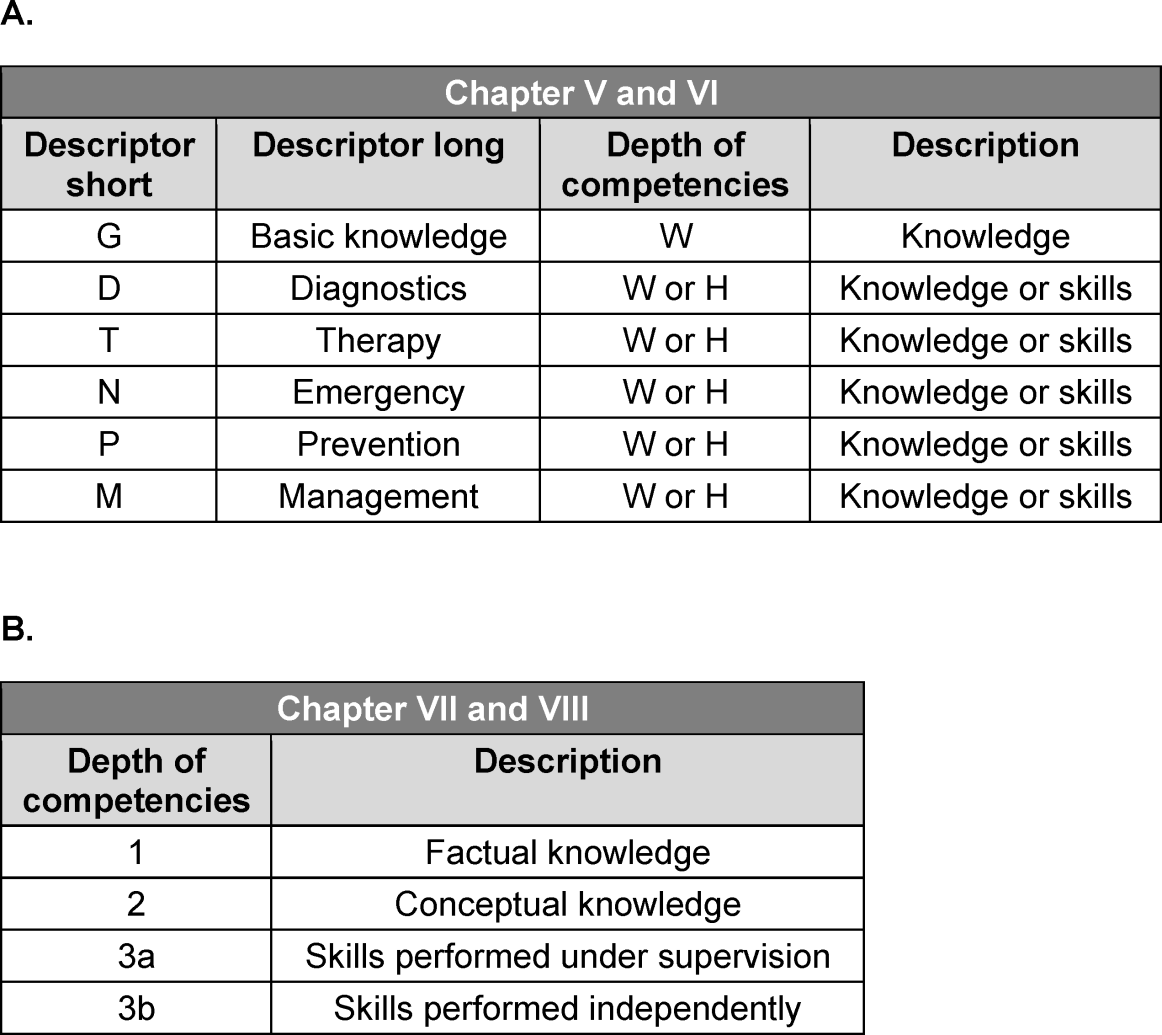
Descriptors and depth of competencies used in the National Competence-Based Catalogue of Learning Objectives 2.0 (NKLM 2.0). There are two different systems, one for chapters V and VI (A) and one for chapters VII and VIII (B). The letter “W” (German: Wissen) indicates knowledge, and the letter “H” (German: Handlungskompetenz) indicates skills.

**Table 2:**
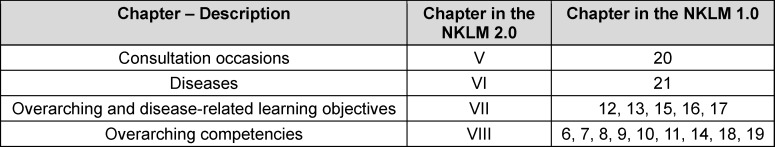
Overview of the changes to the structure of the revised National Competence-Based Catalogue of Learning Objectives (NKLM). Shown are the corresponding chapters in the NKLM 1.0 and the NKLM 2.0.

**Table 3:**
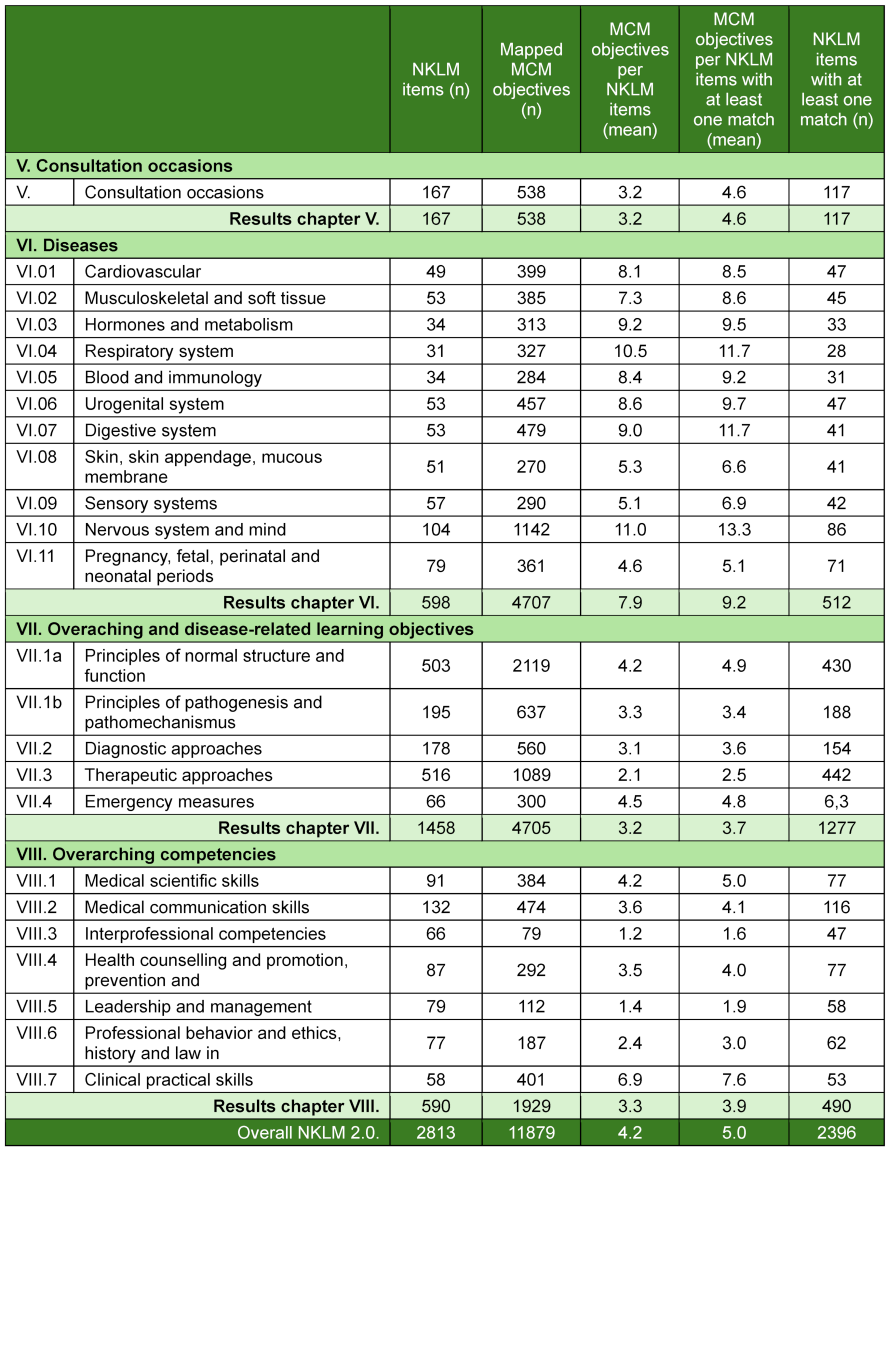
Absolute (n) and mean values (mean) in the mapping of the Modular Curriculum of Medicine (MCM) learning objectives to the items of the National Competence-Based Catalogue of Learning Objectives 2.0 (NKLM).

**Table 4:**
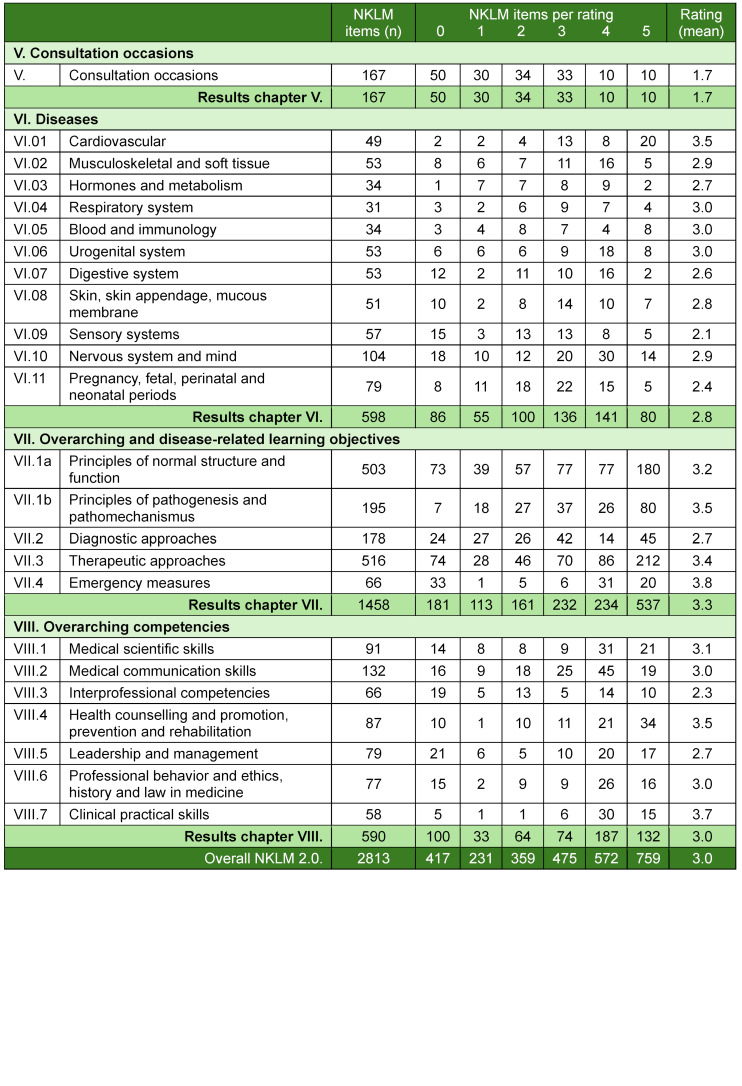
Absolute and mean values for the coverage ratings of the National Competence-Based Catalogue of Learning Objectives 2.0 (NKLM) items by Modular Curriculum of Medicine (MCM) learning objectives based on the qualitative, content comparison approach. The ratings range from 0 (no coverage) to 5 (>80-100% coverage) and represent the relative coverage in percentage form.

**Table 5:**
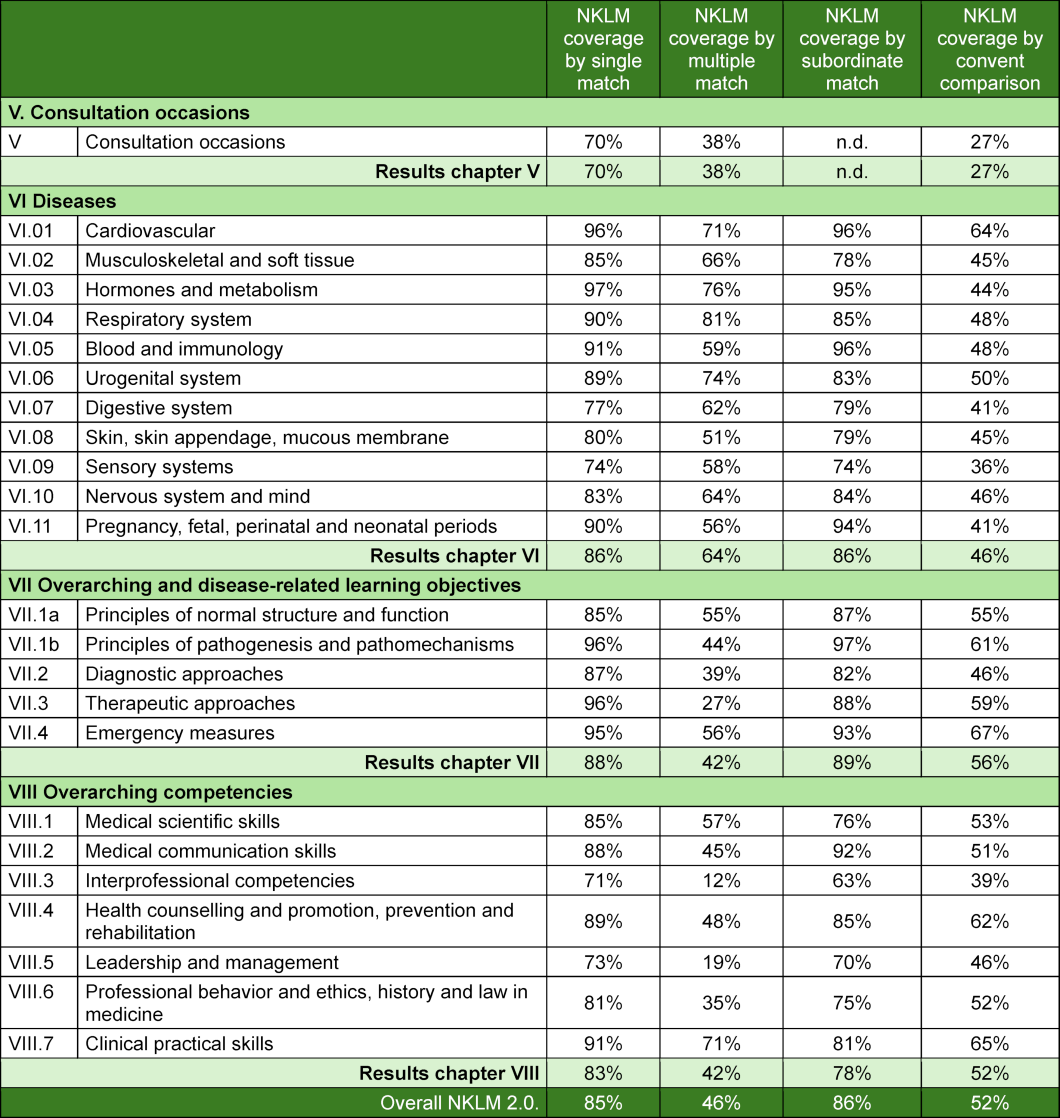
Degree of coverage exhibited by the National Competence-Based Catalogue of Learning Objectives 2.0 (NKLM) overall and by chapter based on mapping of the Modular Curriculum of Medicine (MCM) learning objectives across the four mapping approaches: each single match, multiple match, subordinate match and the qualitative content comparison approach (n.d.=not determined).

**Table 6:**
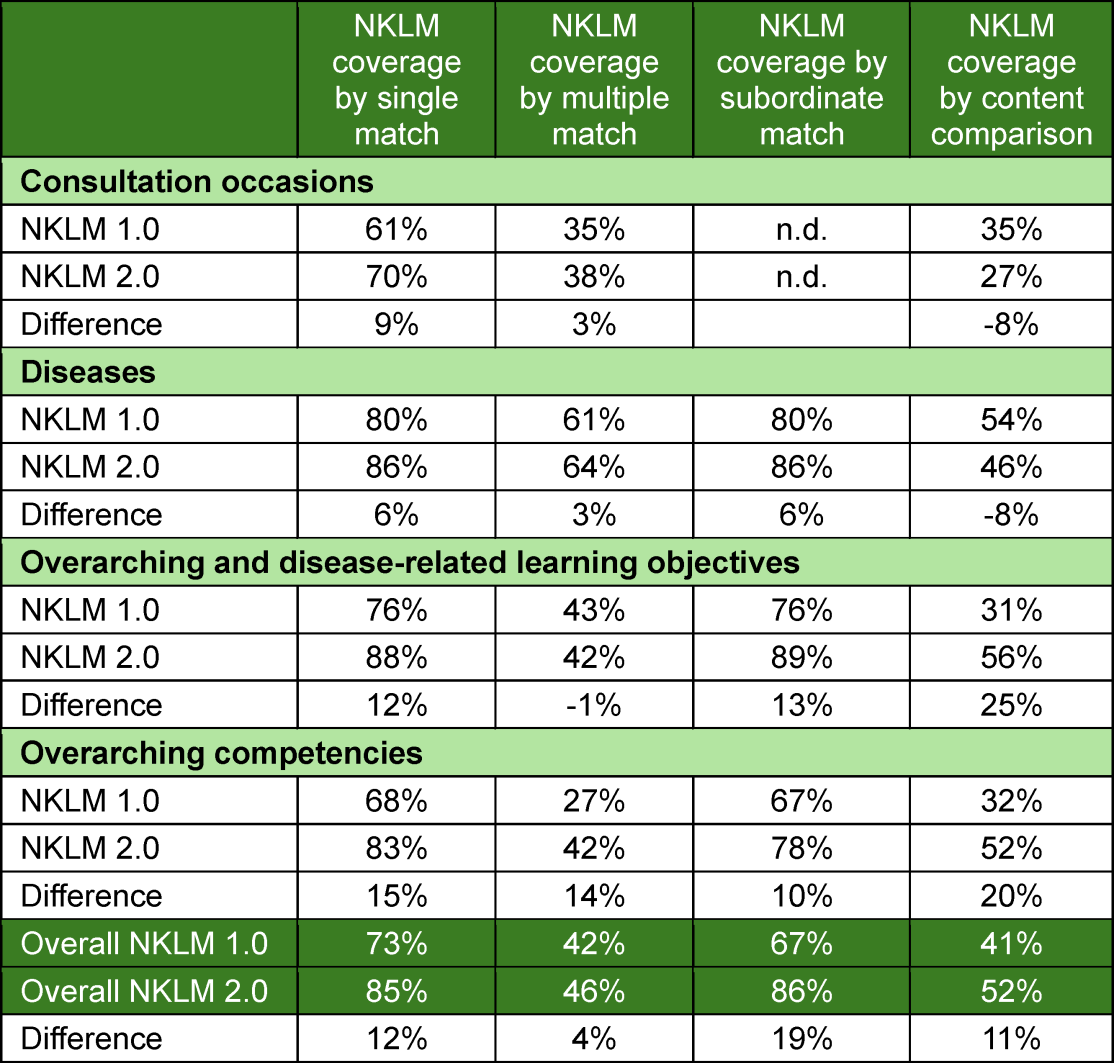
Comparison of the degree of coverage exhibited by the National Competence-Based Catalogue of Learning Objectives 1.0 (NKLM 1.0) and NKLM 2.0 by mappings of the Modular Curriculum of Medicine (MCM) learning objectives across the four different mapping approaches. The results are presented according to the NKLM 2.0 structure, as outlined in table 2.
