# Supplementary material for: Mapping the undergraduate medical curriculum of the Charité Berlin against the National Competence-Based Catalogue of Learning Objectives (NKLM 2.0)
Source: GMS J Med Educ. 2025 Sep 15;42(4):Doc46. doi: 10.3205/zma001770 (PMC12527392; doi:10.3205/zma001770)
Supplement: Illustration of the quantitative mapping approaches [file JME-42-46-s-001.pdf]

Attachment 1: Illustration of the quantitative mapping approaches

1. Single match:

One National Competence-Based Catalogue of Learning Objectives 2.0 (NKLM) item was considered covered if at least one Modular Curriculum of Medicine (MCM) objective was assigned to this category.

For example, 6 out of 8 NKLM items are assigned at least one MCM objective, yielding a coverage of **75%**.

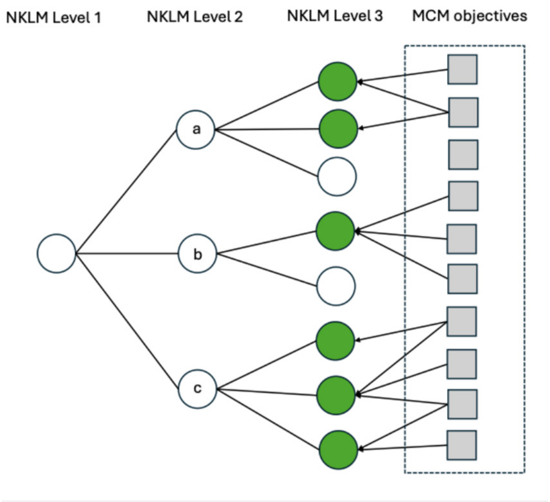

2. Multiple match:

One NKLM item was considered covered if at least three MCM objectives were assigned to this category.

For instance, 2 out of 8 NKLM items are covered by at least three MCM objectives, indicating a **25%** coverage.

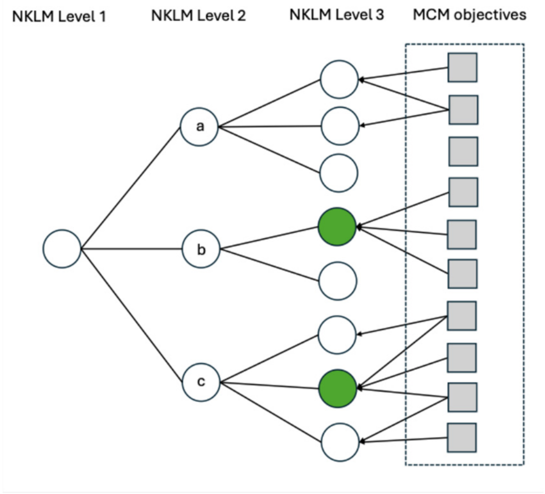

3. Subordinate match:

The coverage of an NKLM objective at level 2 was derived according to the percentage of its subordinate objectives (level 3) that were considered to be covered according to the single match method.

For instance, NKLM Level 2 ‘a’ is covered by 2 of 3 items (67%). ‘b’ by 1 of 2 items (50%) and ‘c’ by 3 out of 3 items (100%). Resulting in a coverage of **72%** (mean of 67%, 50% and 100%).

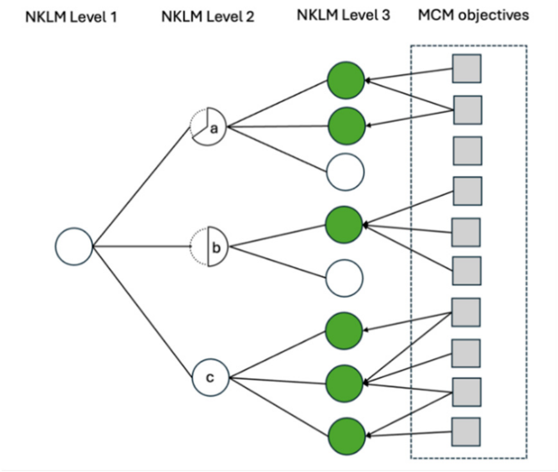

Attachment 1 to Theurich T, Holzhausen Y, Ahlers O, Peters H. *Mapping the undergraduate medical curriculum of the Charité Berlin against the National Competence-Based Catalogue of Learning Objectives (NKLM 2.0).* GMS J Med Educ. 2025;42(4):Doc46. DOI: 10.3205/zma001770
